# A Study Review of the Appropriateness of Oral Antibiotic Discharge Prescriptions in the Emergency Department at a Rural Hospital in Mississippi, USA

**DOI:** 10.3390/antibiotics12071186

**Published:** 2023-07-14

**Authors:** Giang Le, Madalyn Ivy, Sharon Dickey, Ron Welch, Danielle Stallings

**Affiliations:** Department of Pharmacy, Baptist Memorial Hospital—Golden Triangle, Columbus, MS 39705, USA

**Keywords:** antimicrobial stewardship, antibiotic, emergency department, discharge prescriptions, pharmacist

## Abstract

Antimicrobial therapy in emergency departments (EDs) is usually empiric in nature. Due to workload and a goal to reduce patient wait times, providers often make rapid decisions regarding antibiotic prescriptions for discharge. A review of current empiric prescribing practices would determine the appropriateness of oral antibiotic discharge prescriptions from EDs. A single-center retrospective electronic health record review of all adult patients with an ED visit from 1 June 2019, to 30 June 2021 who received at least one oral antibiotic prescription at discharge from Baptist Memorial Hospital-Golden Triangle was conducted. The primary outcome was the assessment of appropriate antibiotic discharge prescriptions. The parameters for appropriateness included empiric drug selection, dosage, frequency, duration, and subsequent cultures and sensitivities. Of the 18,289 identified records, 421 patients were randomly sampled with 400 patients included in the final analysis. Of these, 190 (47.8%) discharge oral antibiotic prescriptions were assessed as appropriate and 209 (52.3%) discharge oral antibiotic prescriptions were assessed as inappropriate based on the guideline recommendations. With approximately half of the patients receiving discharge antibiotics that did not fully follow the guideline recommendations, there is a need for provider education, pharmacist intervention, and antimicrobial stewardship programs focusing on this practice.

## 1. Introduction

Antimicrobial therapy in emergency departments (EDs) is usually empiric therapy. Because of the time constraints, workload, and goal to reduce patient wait times, providers often make rapid decisions on antibiotic prescriptions for discharge. The reported rate of inappropriate antimicrobial use in EDs is similar to the inpatient setting, which is approximately 40–60%, yet the ED has not received as much focus as inpatient care in terms of antimicrobial stewardship interventions [1,2]. In February 2021, the American Society of Health System Pharmacists (ASHP) updated their guidelines for Emergency Department Pharmacy Services, stating that Emergency Medicine Pharmacists (EMPs) can play a role in facilitating the communication between the patient, the outpatient pharmacy, and the ED providers [3]. While there are recommendations from ASHP to utilize pharmacy services, we lack studies that address an antimicrobial stewardship program and the utility of pharmacy services in EDs. It has been shown through a few studies that one can decrease inappropriate drug regimens, reduce readmissions for the same diagnosis, and/or provide optimized patient care by having an EMP review ED discharge prescriptions [4,5,6,7,8,9,10,11,12,13,14,15]. However, these studies’ interventions primarily involved the addition of the EMP to post-discharge culture reviews but there was no review by a pharmacist previously. There is a paucity of studies that investigate pre-discharge antimicrobial stewardship interventions. With our observations that empiric antibiotics can be inappropriate, EDs can benefit from studies that review pre-discharge antibiotic selections.

Baptist Memorial Hospital-Golden Triangle serves the region as a level III trauma center. Our ED has 38 beds and serves approximately 67,000 patients annually from Lowndes County and the surrounding tri-counties. The EMP service was initiated in September 2019. In April 2020, the EMPs’ role expanded to include post-discharge culture reviews. The daily responsibilities of EMPs include reviewing post-discharge cultures that require interventions and providing recommendations to the ED providers based on the microorganism’s sensitivities. Through culture reviews, the EMPs have observed that there are occasions where empiric antibiotic selections were not the drug of choice or were incorrectly dosed. Due to the time it takes for cultures to result, patients may have been on this therapy for a few days prior to a healthcare professional’s intervention. With this observation, we designed a study to investigate the appropriateness of oral antibiotic discharge prescriptions in our ED. A retrospective review of the appropriateness of empiric antibiotic choice, dose, duration, and follow up will be beneficial to our patients to ensure that they receive quality care. This study supports our argument that further studies are needed to identify the need for educational tools and/or expansion of pharmacy services to include collaborative protocols, modification of order sets, or pharmacy consultation of antibiotic selection upon discharge to potentially improve overall patient care.

## 2. Results

**Figure 1:**
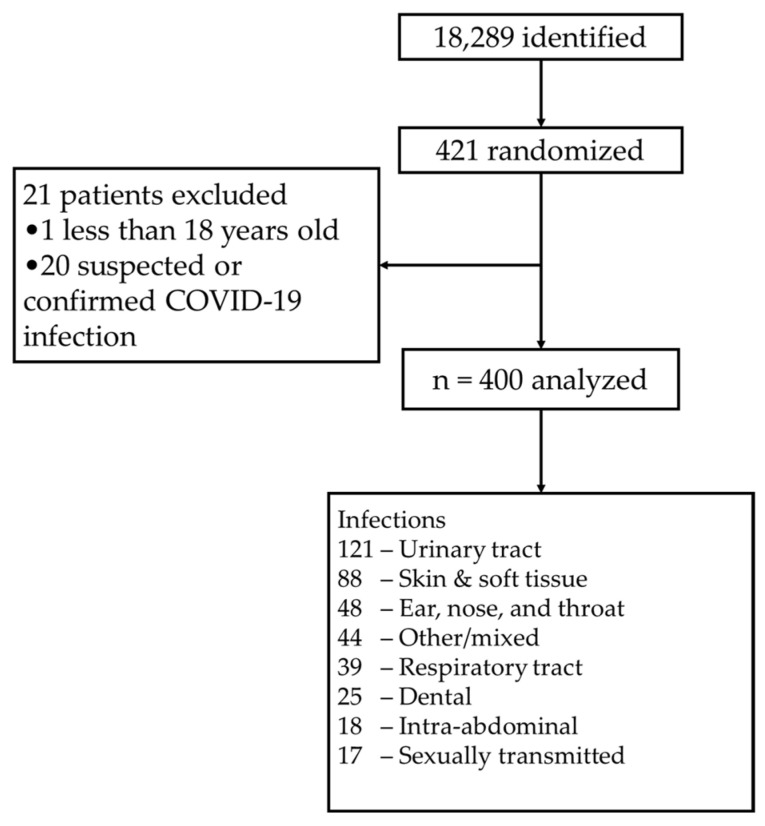
Screening and inclusion/exclusion analysis.

A total of 18,289 records were identified for patients who received at least one oral antibiotic at discharge. Four hundred and twenty-one patients were randomly sampled from the identified records. Twenty-one patients were excluded, with the majority having suspected or confirmed COVID-19 infection. Four hundred patients were included in the final analysis (Figure 1). Table 1 describes the patient demographics. Of the 400 charts reviewed for antibiotic prescriptions at discharge, 191 (47.8%) were assessed as appropriate and 209 (52.3%) were assessed as inappropriate based on the guideline recommendations. Table 2 details the proportions of appropriateness for the specific diagnoses.

Urinary tract infection (30.5%) was the most common indication for discharge antibiotics, followed by skin and soft tissue infections (22.0%), and ear, nose, and throat (ENT) infections (12.0%). The remaining infectious disease (ID) diagnoses included other or mixed, respiratory tract, dental, intra-abdominal, and sexually transmitted infections. The breakdown of ID indications for discharge antibiotics is listed in Table 3.

The most common reasons for an antibiotic prescription being assessed as inappropriate were choice of therapy (34.3%) and duration (28.6%). The remaining reasons for being assessed as inappropriate included dosing (21.9%) and not indicated (15.2%). Inappropriate dosing refers to incorrect dosage strength, incorrect frequency, or absence of renal dose adjustment. Table 4 describes the reasons antibiotic prescriptions were assessed as inappropriate for each ID diagnosis.

Duration and choice of therapy were the most common reasons trimethoprim-SMX (TMP-SMX) was the most inappropriately prescribed antibiotic in the ED. Following TMP-SMX in having the most inappropriate prescriptions were amoxicillin, cephalexin, and azithromycin with dosing and choice of therapy being the most common reasons. Macrobid prescriptions also did not follow guideline recommendations with the duration of therapy being the primary reason. Table 5 describes in greater detail a list of all of the antibiotics that were prescribed inappropriately along with the reasons each agent was given this assessment.

Subgroup analyses based on age and creatinine clearance demonstrated no differences among the groups in terms of incidence of inappropriate prescriptions (see Table 6).

## 3. Discussion

Overall, 52.3% of the antibiotic prescriptions at discharge from the ED did not fully follow guideline recommendations. The results of this study demonstrated that current prescribing practices at our ED can be improved. We identified several areas for targeted interventions (see Table 4 and Table 5). 

For example, 64% of the prescriptions for urinary tract infections were inappropriate due to unnecessarily extended length of treatment. Macrobid prescribed for uncomplicated cystitis was often ordered for 10 days even though the IDSA recommendation is 5 days [16,17]. Similarly, TMP-SMX was frequently prescribed for 10 days for uncomplicated cystitis, as opposed to 3 days as per the guidelines. This increased exposure to antibiotics can lead to adverse events, antimicrobial resistance, and increased cost of treatment [18,19].

Another area of concern was the choice of therapy, which accounted for approximately 50% of the inappropriate prescriptions for skin and soft tissue, ENT, and mixed infections. For skin and soft tissue infections, we found that TMP-SMX was frequently prescribed for cellulitis. For the majority of these cases, penicillins or first-generation cephalosporins would have been adequate to treat cellulitis as per the IDSA recommendations [20]. It should also be noted that in the diagnosis of abscesses, antibiotics may not be necessary if it was a mild infection and if incision and drainage was performed. However, with the limitations of chart reviews, it can be difficult to obtain a complete presentation to assess severity and the need for antibiotics. Due to this reason, treatment after incision and drainage was rarely disagreed upon by the panel. 

In ENT infections, we noticed that azithromycin accounted for 5 out of the 17 patients assessed as receiving an inappropriate choice of therapy. These five patients were diagnosed with sinusitis, tonsillitis, and pharyngitis, and had no known beta-lactam allergy. The guidelines do not recommend azithromycin in patients without a beta-lactam allergy due to the increasing rate of resistance among *Streptococcus pneumonia* [21]. Similarly, of the seven patients diagnosed with otitis media, only one patient was discharged with a first-line option of amoxicillin-clavulanate (four patients were prescribed amoxicillin, one patient was prescribed levofloxacin, and one patient was prescribed TMP-SMX). It should be noted that amoxicillin is not recommended over amoxicillin-clavulanate due to the potential of beta-lactamase-producing *Haemophilus influenzae* or *Moxarella catarrhalis*, which are increasing in prevalence [22]. Amoxicillin-clavulanate is the preferred choice of therapy for bacterial rhinosinusitis [23]. However, due to the limitations of chart reviews, it is often difficult to differentiate viral rhinosinusitis from bacterial rhinosinusitis. For this reason, antibiotics were rarely concluded as not indicated because the panel often had to go by the diagnoses rather than the patients’ presentations. Even though choice of therapy accounted for most prescriptions assessed as inappropriate for the ENT group, the sample size for this group was relatively small and may not accurately represent the population. 

Finally, we identified a prescribing pattern in respiratory tract infections. For this group, 65% of these prescriptions were not indicated. This was primarily due to antibiotics being prescribed for asthma, chronic obstructive pulmonary disease (COPD), and bronchitis. These patients often presented without documented symptoms requiring antibiotics. For example, patients with COPD should have three cardinal symptoms: increase in dyspnea, sputum volume, and sputum purulence; or they should have two of the cardinal symptoms if increased sputum purulence is one of the two symptoms [24]. If these signs and symptoms were not noted in the patient’s chart, the prescription was assessed as not indicated for his or her diagnosis. Similarly, patients presenting with asthma should be assessed for fever, purulent sputum, or radiographic evidence of pneumonia, and other signs and symptoms of infection [25,26]. If the patients did not present with these symptoms, antibiotics should not be prescribed.

One strength of our study was the inclusion of a panel of pharmacists to minimize personal bias from one evaluator. When there was disagreement among the panel, the primary investigator and the co-investigator reviewed the data and strictly adhered to guideline recommendations. As with any study, there were limitations. This was a retrospective chart review with a limited amount of information and the possibility of incomplete documentation. There were variations in provider clinical knowledge and experience, which may affect their prescribing patterns and clinical judgment. The panel assessing the adherence to the guideline recommendations did not include a physician or an infectious disease specialist that could have provided additional perspectives. This was initially considered, but we do not have an infectious disease specialist on staff.

## 4. Materials and Methods

### 4.1. Trial Design and Oversight

This was a single-center, retrospective, electronic health record review of all adult patients with an ED visit from 1 June 2019, to 30 June 2021 who received at least one oral antibiotic prescription at discharge. Appropriateness of discharge antibiotic prescriptions was assessed by a panel of pharmacists including the primary investigator, current ED pharmacist (co-investigator), a pharmacist with ED experience, and a board-certified infectious disease pharmacist. The study was approved by the Baptist Memorial Hospital Institutional Review Board. Due to the study being retrospective in nature, the Institutional Review Board concluded that the study design was ethical as this study did not affect patient care. The first author prepared all drafts of the manuscript. All authors reviewed the manuscript and attested to the accuracy and completeness of the data, the fidelity of the review to the protocol, and accurate reporting of adverse events.

### 4.2. Study Outcome

The primary outcome of this study was the assessment of appropriateness of antibiotic discharge prescriptions. Prescription appropriateness was assessed based on guidelines from Infectious Diseases Society of America (IDSA) [16,17,20,21,23,27,28,29], American Dental Association (ADA), American Gastroenterological Association (AGA), Global Initiative for Chronic Obstructive Lung Disease (GOLD), and Global Initiative for Asthma (GINA). The parameters for assessment of appropriateness included empiric drug selection, dosage, frequency, and duration.

### 4.3. Study Population

The study included all patients 18 years of age and older who received at least one oral antibiotic prescription at discharge from the ED from 1 June 2019, to 30 June 2021. Patients were excluded if they were inmates, admitted to the hospital from the ED visit, transferred to another facility, or discharged with topical antibiotics only, including otic and ophthalmic antibiotics. Patients were also excluded if there was suspected or confirmed COVID-19 infection due to the lack of expert guidance and concerns for superficial infections.

### 4.4. Data Collection and Statistical Analysis

The patients were randomly selected by utilizing an excel formula to divide patients based on disease state. The electronic health records of identified patients were reviewed. The data collected included patient demographics, ICD-10 diagnoses, antibiotics received in the ED, antibiotic discharge prescriptions (agent, dose, frequency, duration of therapy), prescribing provider, any documented adverse events, cultures (microorganisms and sensitivities), laboratory results, pertinent imaging studies, and review of system and physical exams. The patient demographics included patient age, gender, ethnicity, weight, height, body mass index, past medical history, past surgical history, social history, pregnancy status, nursing home resident, allergies, renal function, recent antibiotic use, recent hospitalizations, presence of catheters or drains, and vital signs. The data were analyzed using descriptive statistics for continuous and nominal data. For a 95% confidence interval and a 5% margin of error in determining appropriateness, we calculated that we needed a total of 377 patients to determine power.

## 5. Conclusions

Based on our results, we have identified several areas in which our ED can improve discharge antibiotic prescription selection, enhance overall antimicrobial stewardship, and provide a higher quality of care for our patients. First, education will be provided to improve providers’ prescribing patterns. Potential interventions include providing education to the providers through in-services or pocket cards providing updates and guidelines for antibiotic selection for each individual infection. There are some interventions that can be implemented to assist with more appropriate prescribing. For instance, we can improve the providers’ selection and dosing by adjusting the electronic health system orders. Order panels built for specific infection diagnoses can be an option to ensure that first-line agents are used and dosed appropriately. Secondly, we identified that TMP-SMX was the most common antibiotic that is not renally adjusted [30]. By adding renal dosing information at the time of decision making, this will aid in more appropriate dosing and avoid medication errors. Finally, inclusion of the ED pharmacists at the time of decision making could ensure goal-directed therapy and aid with renal dose adjustments. The electronic health record could provide a notification system for pharmacy to immediately review discharge antibiotic prescriptions. This could also be managed by consulting the pharmacist or by pharmacy protocol or collaborative agreement with the ED providers. 

## Figures and Tables

**Table 1:** Demographic data of study population.

Demographics	*n* = 400
Average Age—years	42.2 ± 19.6
Age	
≥65 years	67 (16.8%)
18–64 years	333 (83.3%)
Female	276 (69.0%)
Race	
Caucasian	142 (35.5%)
African American	253 (63.3%)
Other	5 (1.3%)
Average BMI—kg/m^2^	31.4 ± 9.1
Creatinine Clearance	
>60 mL/min	140 (35.0%)
30–60 mL/min	43 (10.8%)
<30 mL/min	19 (4.8%)
No lab	198 (49.5%)
Temperature	
≥100.4 °F	15 (3.8%)
<100.4 °F	384 (96.0%)
Not recorded	1 (0.3%)
White Blood Cell Count	
>12,000 cell/mm^3^	43 (10.8%)
4000–12,000 cell/mm^3^	161 (40.3%)
<4000 cell/mm^3^	3 (0.8%)
No lab	193 (48.3%)
Beta-Lactam Allergy	73 (18.3%)

**Table 2:** Appropriateness of antibiotic discharge prescriptions by indication.

Infection	Appropriate	Inappropriate
Overall	191 (47.8%)	209 (52.3%)
Urinary tract	61 (50.0%)	61 (50.0%)
Skin and soft tissue	50 (56.8%)	38 (43.2%)
Ear, nose, and throat	15 (31.3%)	33 (68.8%)
Other or mixed	25 (56.8%)	19 (43.2%)
Respiratory tract	13 (33.3%)	26 (66.7%)
Dental	11 (44.0%)	14 (56.0%)
Intra-abdominal	9 (55.6%)	8 (44.4%)
Sexually transmitted	7 (41.2%)	10 (58.8%)

**Table 3:** Infection indications with number of oral antibiotic discharge prescriptions.

Infection	*n*
Urinary tract	122 (30.5%)
Skin and soft tissue	88 (22.0%)
Ear, nose, and throat	48 (12.0%)
Other or mixed	44 (11.0%)
Respiratory tract	39 (9.8%)
Dental	25 (6.3%)
Intra-abdominal	17 (4.3%)
Sexually transmitted	17 (4.3%)

**Table 4:** Reasons antibiotic prescriptions were assessed as inappropriate by indication.

Infection	Total Inappropriate Prescriptions, *n*	Choice of Therapy	Dosing ^a^	Not Indicated	Duration
		*n*	*n*	*n*	*n*
Overall	209	72 (34.3%)	46 (21.9%)	32 (15.2%)	60 (28.6%)
Urinary tract	61	15 (24.6%)	6 (9.8%)	1 (1.6%)	39 (63.9%)
Skin, soft tissue	38	19 (50.0%)	13 (34.2%)	5 (13.2%)	1 (2.6%)
Ear, nose, throat	33	17 (51.5%)	11 (33.3%)	2 (6.1%)	3 (9.1%)
Other or mixed	19	9 (47.4%)	4 (21.1%)	2 (10.5%)	4 (21.1%)
Respiratory tract	26	7 (26.9%)	0	17 (65.4%)	2 (7.7%)
Dental	14	1 (7.1%)	8 (57.1%)	0	5 (35.7%)
Intra-abdominal	8	2 (25.0%)	3 (37.5%)	3 (37.5%)	0
Sexually transmitted	10	1 (10.0%)	1 (10.0%)	1 (10.0%)	7 (70.0%)

^a^ Dosing includes incorrect dosage strength, incorrect frequency, and/or dosing was not renally adjusted.

**Table 5:** Reasons antibiotic prescriptions were assessed as inappropriate by agent.

Agent	Total Inappropriate Prescriptions, *n*	Choice of Therapy	Dosing ^b^	Not Indicated	Duration
		*n*	*n*	*n*	*n*
Amoxicillin	27	6 (22.2%)	12 (44.4%)	3 (11.1%)	6 (22.2%)
Amoxicillin-Clavulanate	4	1 (25.0%	0	0	3 (75.0%)
Azithromycin	25	12 (48.0%)	0	13 (52.0%)	0
Cefdinir	3	0	1 (33.3%)	0	2 (66.7%)
Cefprozil	1	0	1 (100.0%)	0	0
Cefuroxime	2	1 (50.0%)	1 (50.0%)	0	0
Cephalexin	26	7 (26.9%)	13 (50.0%)	4 (15.4%)	2 (7.7%)
Ciprofloxacin	12	1 (8.3%)	0	1 (8.3%)	10 (83.3%)
Clindamycin	16	7 (43.8%)	7 (43.8%)	1 (6.2%)	1 (6.2%)
Doxycycline	10	3 (30.0%)	1 (10.0%)	5 (50.0%)	1 (10.0%)
Levofloxacin	7	5 (71.4%)	1 (14.3%)	1 (14.3%)	0
Macrobid	17	5 (29.4%)	0	1 (5.9%)	11 (64.7%)
Macrodantin	3	3 (100.0%)	0	0	0
Metronidazole	17	3 (17.6%)	6 (35.3%)	1 (5.9%)	7 (41.2%)
Penicillin VK	2	1 (50.0%)	1 (50.0%)	0	0
Rifampin	2	2 (100.0%)	0	0	0
Trimethoprim-SMX	35	14 (40.0%)	2 (5.7%)	1 (2.9%)	18 (51.4%)

^b^ Dosing includes incorrect dosage strength, incorrect frequency, and/or dosing was not renally adjusted.

**Table 6:** Subgroup analyses of patients with assessment of inappropriate antibiotic discharge prescriptions.

Subgroup	Inappropriate Prescriptions
Age	
≥65 years	38 (56.7%)
18–64 years	172 (51.7%)
Creatinine Clearance	
>60 mL/min	68 (48.6%)
30–60 mL/min	23 (53.5%)
<30 mL/min	10 (52.6%)
No lab	109 (55.1%)
Provider	
Physicians	88 (50.9%)
Nurse Practitioners	107 (56.3%)
Physician Assistants	15 (40.5%)

## Data Availability

The data presented in this study are available on request from the corresponding author.
